# REX-1 Expression and p38 MAPK Activation Status Can Determine Proliferation/Differentiation Fates in Human Mesenchymal Stem Cells

**DOI:** 10.1371/journal.pone.0010493

**Published:** 2010-05-05

**Authors:** Dilli Ram Bhandari, Kwang-Won Seo, Kyoung-Hwan Roh, Ji-Won Jung, Soo-Kyung Kang, Kyung-Sun Kang

**Affiliations:** 1 Adult Stem Cell Research, College of Veterinary Medicine, Seoul National University, Seoul, Republic of Korea; 2 Laboratory of Stem Cell and Tumor Biology, Department of Veterinary Public Health, College of Veterinary Medicine, Seoul National University, Seoul, Korea; 3 Laboratory of Veterinary Biotechnology, College of Veterinary Medicine and BK21 Program for Veterinary Science, Seoul National University, Seoul, Korea; University of Southern California, United States of America

## Abstract

**Background:**

REX1/ZFP42 is a well-known embryonic stem cell (ESC) marker. However, the role of REX1, itself, is relatively unknown because the function of REX1 has only been reported in the differentiation of ESCs via STAT signaling pathways. Human mesenchymal stem cells (hMSCs) isolated from young tissues and cancer cells express REX1.

**Methodology/Principal Finding:**

Human umbilical cord blood-derived MSCs (hUCB-MSCs) and adipose tissue-derived MSCs (hAD-MSCs) strongly express REX1 and have a lower activation status of p38 MAPK, but bone marrow-derived MSCs (hBM-MSCs) have weak REX1 expression and higher activation of p38 MAPK. These results indicated that REX1 expression in hMSCs was positively correlated with proliferation rates but inversely correlated with the phosphorylation of p38 MAPK. In hUCB-MSCs, the roles of REX1 and p38 MAPK were investigated, and a knockdown study was performed using a lentiviral vector-based small hairpin RNA (shRNA). After REX1 knockdown, decreased cell proliferation was observed. In REX1 knocked-down hUCB-MSCs, the osteogenic differentiation ability deteriorated, but the adipogenic potential increased or was similar to that observed in the controls. The phosphorylation of p38 MAPK in hUCB-MSCs significantly increased after REX1 knockdown. After p38 MAPK inhibitor treatment, the cell growth in REX1 knocked-down hUCB-MSCs almost recovered, and the suppressed expression levels of CDK2 and CCND1 were also restored. The expression of MKK3, an upstream regulator of p38 MAPK, significantly increased in REX1 knocked-down hUCB-MSCs. The direct binding of REX1 to the *MKK3* gene was confirmed by a chromatin immunoprecipitation (ChIP) assay.

**Conclusions/Significance:**

These findings showed that REX1 regulates the proliferation/differentiation of hMSCs through the suppression of p38 MAPK signaling via the direct suppression of MKK3. Therefore, p38 MAPK and REX-1 status can determine the cell fate of adult stem cells (ASCs). These results were the first to show the role of REX1 in the proliferation/differentiation of ASCs.

## Introduction

Embryonic stem cells (ESCs) are pluripotent stem cells that can self-renew and generate all the cell types of the body; however, they are not able to generate the extra embryonic trophoblast lineage [1]. The transcriptional regulatory network of ESCs that maintains pluripotency is well-established. Takahashi and Yamanaka reported critical transcription factors that are necessary for the induction of pluripotency [2]. The core transcription factors, including the Yamanaka factors, have been relatively well-defined in ESCs [3], [4]. OCT4 [5] and REX1 [6] are transcription factors that are characteristic markers of pluripotent stem cells. Paradoxically, over- or under-expression of Oct4 leads to the down-regulation of Rex1 expression. Down-regulation of Oct4 and Rex1 triggers trophectoderm differentiation, while their up-regulation triggers primitive endoderm and mesoderm differentiation [7]. *Rex1* (Zfp42) was first identified as a gene that is transcriptionally repressed by retinoic acid and encodes a zinc finger transcription factor that is expressed at high levels in F9 teratocarcinoma stem cells, embryonic stem cells, and other stem cells [8]–[10]. REX1 is a member of the YY1 sub-family of transcription factors that can function as repressors, activators or transcription initiators depending on the sequence context of the YY1-binding sites with respect to other regulatory elements [9],[11]. Currently, REX1 is widely used as a stem cell marker, and Rex1 inhibits signaling via the Janus kinase (JAK)/STAT3 pathway during the differentiation of F9 teratocarcinoma stem cells [12]. ESCs from Rex1 knock-out mice show defects in the induction of a subset of marker genes in the visceral endoderm, which suggests that Rex1 plays a role in ESC differentiation [13].

The family of Mitogen-Activated Protein Kinases (MAPKs) controls an enormous number of processes such as gene expression, metabolism, cell proliferation, division, differentiation, apoptosis and embryogenesis [14], [15]. Five different MAPK pathways have been described: the extracellular signal-regulated kinases (ERKs), the stress-activated protein kinases (SAPKs), the c-Jun N-terminal kinases (JNK), the ERK5/big MAP kinase 1 (BMK 1) and the p38 MAPK. The p38 MAPK pathway was initially described as being activated by different types of cellular stresses and cytokines. Numerous studies have reported the involvement of p38 MAPK pathways in the regulation of a wide spectrum of cellular processes including cell cycle arrest, apoptosis, senescence, regulation of RNA splicing, tumorigenesis and the growth/differentiation of specific cell types [16], [17]. In mammals, there are four p38 MAPKs: p38α, p38β, p38γ (SAPK3, ERK6) and p38δ (SAPK4). MAP kinase p38α is ubiquitously expressed whereas p38β, p38γ and p38δ have restricted expression patterns [18]. Two major MAPK kinases (MKKs), MKK3 and MKK6, are known to activate p38 MAPKs. MKK6 activates all four p38 MAPKs and MKK3 activates p38α, p38γ and p38δ [17], [19].

Mesenchymal stem cells (MSCs) are promising tools in the field of regenerative medicine. MSCs have been isolated from bone marrow, adipose tissue, peripheral blood, fetal liver, lung, amniotic fluid, chorionic villi of the placenta and umbilical cord blood [20]–[25]. However, their ability to proliferate and differentiate differs depending on their parental tissue type and subsequent culture conditions. Roch et al. [26] described that OCT4, REX1 and GATA4 expression in human MSCs increases the differentiation efficiency of these cells. Furthermore, first-trimester human fetal MSCs express OCT4, NANOG and REX1 [27]; therefore, hMSCs originating from young tissue have a strong potential to obtain powerful multipotency and become large cell populations. In addition to the isolation method, the culturing method is another challenge in stem cell biology. Inhibition of p38 MAPK facilitates *ex vivo* expansion of skin epithelial progenitor cells [28], and several types of p38 MAPK inhibitors have been reported in the literature. In this report, we determined that the phosphorylation status of p38 MAPK and the expression of REX1 in hMSCs was an important regulatory machine that maintains ASCs via the direct regulation of MKK3.

## Materials and Methods

### Isolation of hMSCs, Cell Culture and Ethics Statement

Human umbilical cord blood-derived MSCs (hUCB-MSCs) [29], human bone marrow derived MSCs (hBM-MSCs) [30] and human adipose tissue-derived MSCs (hAD-MSCs) [31] were isolated and cultured as previously described. In brief, two clones of hAD-MSCs were isolated from freshly excised mammary fat tissue acquired from the Ba-Ram plastic surgery hospital. Tissues were obtained from 20 to 30 year-old women during reduction mammoplasty. The hAD-MSCs were maintained in K-SFM medium supplemented with 2 mM N-acetyl-L-cysteine (Sigma-Aldrich, St. Louis, MO, USA) and L-ascorbic acid (0.2 mM, Sigma-Aldrich). hBM-MSCs were isolated from three healthy donors and were cultured in low glucose Dulbecco's Modified Eagle's Medium (DMEM) supplemented with 10% fetal bovine serum (FBS) without any additional growth factors. hUCB-MSCs were obtained from umbilical cord blood immediately after full term delivery with written consent from 20 to 30 year-old mothers and the approval of the Boramae Hospital Institutional Review Board (IRB). Three hUCB-MSC clones were used in this experiment. The hUCB-MSCs were maintained in DMEM (Invitrogen, Carlsbad, USA) containing 10% FBS. The passages (p) of hMSCs used for the experiments were p5 in hUCB-MSCs, p5 in hAD-MSCs and p3 in hBM-MSCs. The isolation and research use of hAD-MSCs and hBM-MSCs were also approved by the Boramae Hospital IRB with written consent. All procedures were approved by the institutional review board of Seoul National University (UCB-MSC, #0603/001-002; AD-MSC, #0600/001-001; BM-MSC, #0910/001-003).

### Cell proliferation and cell cycle analyses

To measure cell growth, CCK-8 (Dojindo Molecular Technologies Inc., San Diego, CA, USA) was used according to the manufacturer's protocol, and cells were measured at a wavelength of 540 nm in an enzyme-linked immunosorbent assay plate reader (EL800, Bio-Tek Instruments Inc., Winooski, VT, USA).

The stages of the cell cycle were detected by FACS analysis. Briefly, the cells were washed twice with PBS and harvested by trypsinization after 3 days. The cells were then washed again with PBS and fixed with 70% ethanol at −20°C for 1 day. The fixed cells were washed with ice cold PBS and stained with 50 µg/ml of propidium iodide (Sigma, St Louis, Missouri, USA) in the presence of 100 µg/ml RNase A (Sigma) for 30 minutes. The cell cycle stages were analyzed using the FACS Calibur (Becton & Dickinson, NJ, USA).

### The construction and production of lentiviral vectors

Lentiviruses were generated using ViraPower™ Lentiviral packaging Mix (Invitrogen, Carlsbad, CA, USA). Lipofectamine 2000 (Invitrogen) was used for the transfection of 293FT cells (Invitrogen) with SHDNAC-TRCN0000107810 (REX1 knockdown-2, R2), SHDNAC-TRCN0000107812 (REX1 knockdown-4, R4) and SHC002 (VC, random sequence inserted) (Sigma, Saint Louis, MO, USA). Cell culture media was changed the day after transfection and the supernatant was harvested at 48 and 72 hours post transfection. The viral supernatant was filtered using 0.4-µm pore filters (Invitrogen). Cells were transfected with *REX-1* shRNA-producing lentivirus at MOI (multiplicity of infection) of 5–10. Polybrene (Sigma) was added to the cell culture media at a final concentration of 6 µg/ml. The cell culture medium was replaced with fresh culture medium the day after transfection. For selection, puromycin was added to the cell culture media at a final concentration of 3 µg/ml for 3 days.

### RT-PCR and ChIP assay

Total RNA was extracted with an easy-spin™ Total RNA Extraction Kit (iNtRON Biotechnology, Sungnam, Korea) according to the manufacturer's instructions. Synthesis of cDNA was carried out using the SuperScript® III First-Strand Synthesis System for RT-PCR (Invitrogen) with 1 µg total RNA and oligo dT primers. The primers for each gene are shown in Table S1. Gene expression was also analyzed using real-time PCR with SYBR Green Master Mix reagents (Applied Biosystems, Foster City, CA, USA). The expression level of *REX-1* was detected, and the real-time PCR values for gene expression were normalized to Glyceraldehyde 3-phosphate dehydrogenase (GAPDH) expression. Real time RT-PCR was performed with a LightCycler 480 Real-Time PCR System (Roche, Indianapolis, IN, USA). The chromatin immunoprecipitation (ChIP) assay was carried out according to the manufacturer's protocol (cat#17-295, Upstate Biotechnology, Billerica, MA, USA). PCR primers for the ChIP assay are listed in Table S1.

### Immunofluorescence staining

Cells were fixed with 4% paraformaldehyde for 20 minutes at room temperature, and incubated with blocking solution (10% normal goat serum, Rockland Immunochemicals, Gilbertsville, PA, USA) overnight at 4°C. The cells were then incubated overnight at 4°C with REX-1 primary antibody diluted in blocking solution (ab50828, Abcam, Cambridge, MA, USA), following which the cells were treated with the Alexa Fluor anti-rabbit IgG secondary antibody (Invitrogen) for 1 hour. For nuclear counter-staining, Hoechst 33238 (1 µg/ml, Sigma) was diluted to 1∶500 in PBS and incubated with the cells for 15 minutes. Images were captured with a confocal microscope (Eclipse TE200, Nikon, Tokyo, Japan).

### Western blotting

Cells were lysed with PRO-PREP (#17081, iNtRON Biotechnology). The cell lysates were then incubated on ice for 20 minutes followed by centrifugation (13,000 rpm, 15 minutes, 4°C) and supernatant collection. The protein concentrations of samples were determined using the Protein Assay Reagent (Bio-Rad laboratories, Hercules, CA, USA) according to the manufacturer's instructions. The protein samples (10–15 µg) were electrophoresed using a 10–12% SDS-polyacrylamide electrophoresis gel. The proteins were detected with primary antibodies that recognize REX1 (ab50828, Abcam), CDK2 (#2546, Cell signaling Inc, Danvers, MA, USA), CDK4 (#2906, Cell signaling Inc), Cyclin B1 (#4138, Cell signaling Inc), Cyclin D1 (#2926, Cell signaling Inc), p21 (sc-32, Santa Cruz Biotechnology, Santa Cruz, CA, USA), BAX (sc-493, Santa Cruz Biotechnology), pERK1/2 (V803A, Promega, Madison, WI, USA), ERK1/2 (V114A, Promega), pGSK3β (#9336, Cell signaling Inc), GSK3β (#9315, Cell signaling Inc), pSTAT3 (#9131, Cell signaling Inc), STAT3 (#9139, Cell signaling Inc), STAT5 (sc-835, Santa Cruz Biotechnology), MEK1/2 (#9122, Cell signaling Inc), pMEK1/2 (#9121, Cell signaling Inc), NF-κB (#3034, Cell signaling Inc), HES1 (AB5702, Chemicon), Pp38 (#4631, Cell signaling Inc), p38 (#9212, Cell signaling Inc), MKK3 (#9232, Cell signaling Inc) and GAPDH (MAB374, Chemicon). Antibody recognition was detected with the respective secondary antibody linked to horseradish peroxidase (Zymed Laboratories Inc., South San Francisco, CA, USA). Secondary horseradish peroxidase–conjugated antibodies were detected by enhanced chemiluminescence (ImageQuant 400, GE Healthcare, Piscataway, NJ, USA). The relative quantities of each protein band, normalized to control cells, were quantified using Quantity One software (version 4.6.5, Bio-Rad Inc, Hercules, CA, USA).

### ANNEXIN V staining for apoptosis

The apoptosis assay was performed with a two-color analysis of FITC-labeled ANNEXIN V binding and propidium iodide (PI) uptake using the ANNEXIN V–FITC Apoptosis Detection kit following the manufacturer's instructions (Calbiochem, San Diego, CA, USA). Positioning of quadrants on ANNEXIN V/PI dot plots was performed, and live cells (ANNEXIN V^–^/PI^–^), early/primary apoptotic cells (ANNEXIN V^+^/PI^–^), late/secondary apoptotic cells (ANNEXIN V^+^/PI^+^), and necrotic cells (ANNEXIN V^–^/PI^+^) were distinguished.

### Statistical analysis

The data are presented as mean value ± standard deviation obtained from three independent experiments in which hMSCs clones originated from three individuals. The student's *t*-test (Microsoft Excel) was used for statistical analysis. Data are considered statistically significant when p<0.01.

## Results

### The expression of REX1 is negatively correlated with the phosphorylation of p38 MAPK in hMSCs

**Figure 1:**
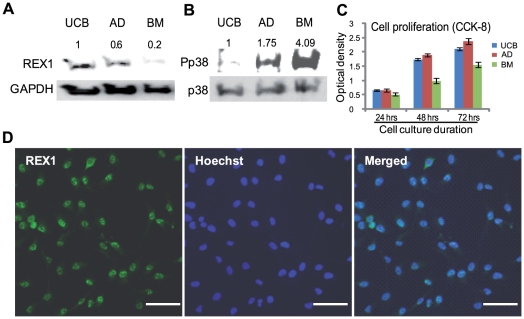
The expression levels of REX1, p38 MAPK and the cell proliferation of hMSCs. (A) The expression of REX1 in hUCB-MSCs, hAD-MSCs and hBM-MSCs. hUCB-MSCs and hAD-MSCs have strong REX1 expression, but hBM-MSCs have very weak REX1 expression. (B) The expression of p38 and phosphorylated p38 (Pp38) in hUCB-MSCs, hAD-MSCs and hBM-MSCs. The expression level of p38 was similar in the three types of hMSCs examined. hBM-MSCs have the strongest expression level of Pp38 among the three types of hMSCs. (C) Cell proliferation measured by CCK-8. The growth of hBM-MSCs was slowest among the three types of hMSCs. (D) REX1 primarily localized in the nucleus of hUCB-MSCs. Scale bars represent 100 µm. Error bars represent the standard deviation from three independent experiments.

REX1 is expressed in ESCs and MSCs. Among three well-characterized hMSCs, REX1 is strongly expressed in hUCB-MSCs and hAD-MSCs; however, REX1 is weakly expressed in hBM-MSCs when grown under previously reported culture conditions [29]–[31]. In hUCB-MSCs, REX1 expression was nearly five-fold greater than in hBM-MSCs (Fig. 1A). However, in three types of hMSCs, the levels of phosphorylated p38 MAPK (Pp38) was in inverse proportion to the expression of REX1 (Fig. 1B). The level of Pp38 in hBM-MSCs was four-fold stronger than the level in hUCB-MSCs. The expression level of p38, itself, was similar between the three types of hMSCs (Fig. 1B). The cell proliferation rates of these three types of hMSCs were quite different. In repeated experiments, hUCB-MSCs and hAD-MSCs grew faster than hBM-MSCs (Fig. 1C). Therefore, the proliferation rates of hMSCs positively correlate with the REX1 expression level but inversely correlate with the level of Pp38 expression. REX1 was expressed in the majority of hUCB-MSCs, and the localization of REX1 was primarily confined to the nucleus of hUCB-MSCs as shown by immunocytochemistry (Fig. 1D).

### Knockdown of REX1 resulted in growth retardation of hUCB-MSCs

**Figure 2:**
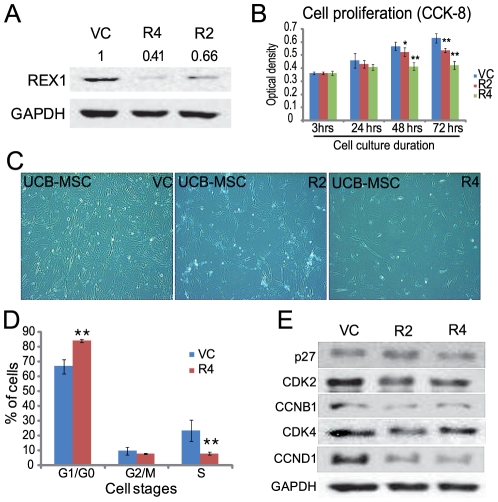
REX1 knockdown resulted in the growth retardation of hUCB-MSCs. (A) The expression of REX1 decreased after REX1-knockdown lentivirus infection. (B) The cell proliferation of REX1 knocked-down hUCB-MSCs significantly decreased from two days after infection. (C) The morphology of cells three days after culturing. REX1 knocked-down cells are not growing at a rate similar to the control cells. (D) Cell cycle arrest was observed in REX1 knocked-down hUCB-MSCs using FACS analysis. (E) The expression levels of Cyclins, CDK and cell cycle inhibitors. The expression levels of CDK2, CCNB1, CDK4 and CCND1 decreased, but p27 did not change after REX1 knockdown. Error bars represent the standard deviation from three independent experiments. *, p<0.05; **, p<0.01.

hUCB-MSCs grew faster than hBM-MSCs but at a similar pace as hAD-MSCs in *in vitro* culture. The discrepancy in the proliferation speed may be potentially due to the difference in REX1 expression in the different cell types. Therefore, a REX1 knockdown experiment was performed in hUCB-MSCs, which had the highest expression of REX1 among the three types of hMSCs. Four different shRNA producing lentiviruses that target different REX1-regions were used for the experiment. Two lentiviruses construct, REX1-2 (R2) and REX1-4 (R4), showed specific inhibition of REX1 after puromycin selection (Fig. 2A). After REX1 knockdown, cell growth severely decreased in hUCB-MSCs (Fig. 2B and 2C). For detailed analysis of cell cycle progression, FACS analysis was performed with the hUCB-MSCs (Fig. 2D). The composition of the G0/G1 phase significantly increased and the composition of the S phase significantly decreased in REX1 knocked-down hUCB-MSCs compared to vehicle control-infected hUCB-MSCs. Western blot analysis of cell cycle regulators was performed using vehicle control-infected and REX1 knocked-down hUCB-MSCs. After REX1 knockdown, the expression levels of CDK2, Cyclin B1, CDK4 and Cyclin D1 decreased compared to the levels of vehicle control-infected hUCB-MSCs. However, the expression of p27 did not change when REX1 was knocked-down in hUCB-MSCs compared to vehicle control-infected hUCB-MSCs (Fig. 2E).

### Phosphorylation of p38 MAPK significantly increased following the knockdown of REX1 in hUCB-MSCs

**Figure 3:**
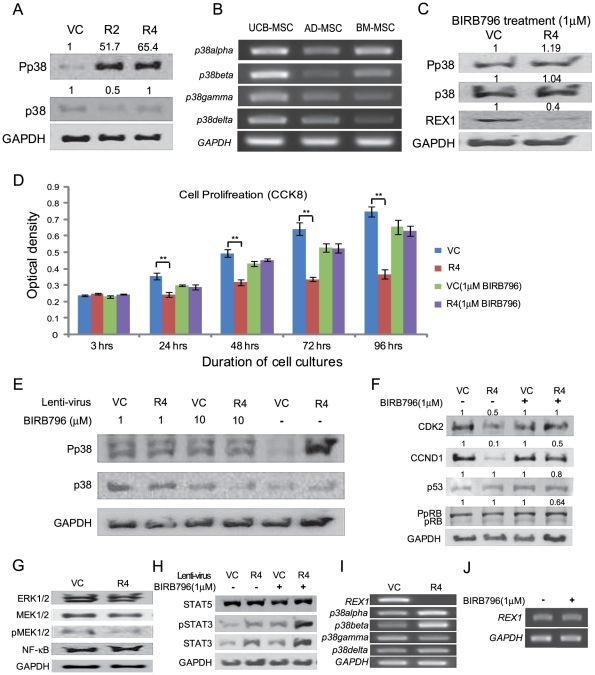
The levels of p38 MAPK, Cyclins and cell cycle inhibitors changed after REX1 knockdown and p38 MAPK inhibitor treatment. (A) Significant phosphorylation of p38 MAPK (Pp38) was seen after REX1 knockdown in hUCB-MSCs. (B) hUCB-MSCs, hAD-MSCs and hBM-MSCs express all four types of p38 isoforms. (C) After p38 MAPK inhibitor treatment (1 µM BIRB796), the phosphorylation of p38 MAPK was similar in REX1 knocked-down hUCB-MSCs compared to vehicle control-infected hUCB-MSCs. (D) Cell proliferation was measured with CCK-8 for four days. The cell proliferation of REX1 knocked-down hUCB-MSCs was similar with vehicle control-infected hUCB-MSCs after BIRB796 (1 µM) treatment. Without BIRB796, the proliferation of REX1 knocked-down hUCB-MSCs significantly decreased compared with vehicle control-infected hUCB-MSCs. **, p<0.01. (E) The p38 MAPK of REX1 knocked-down hUCB-MSCs was not activated after 1 µM or 10 µM BIRB796 treatment compared to those of vehicle control-infected hUCB-MSCs. (F) Changes in the expression of CDK and cell cycle inhibitors. The decreased expression of CDK2 and CCND1 recovered after p38 MAPK inhibitor treatment. (G) The expression levels of ERK1/2, MEK, phospho-MEK (pMEK1/2) and NF-κB did not significantly change after REX1 knockdown in hUCB-MSCs. (H) The expression changes of STAT3 and STAT5. The expression levels of STAT3 and phospho-STAT3 were significantly increased after REX1 knockdown without regard to p38 MAPK inhibitor treatment. STAT5 expression did not change after the knockdown of REX1. (I) p38α and p38β were up-regulated but p38γ was down-regulated after REX1 knockdown in hUCB-MSCs. (J) *REX1* expression of hUCB-MSCs did not change after BIRB796 (p38 MAPK inhibitor) treatment. Error bars represent the standard deviation from three independent experiments.

Among the detected MAPKs in hUCB-MSCs, p38 MAPK signaling significantly changed when REX1 was knocked-down. The expression level of p38 MAPK did not increase after REX1 knockdown; however, the level of Pp38 increased by more than fifty-fold after REX1 was knocked-down in hUCB-MSCs, which was verified in multiple experiments (Fig. 3A). SB203580 and SB202190 are potent inhibitors of the p38α and p38β isoforms, respectively, and all p38 MAPK isoforms are inhibited by BIRB796 [32]. The hUCB-MSCs express all four types of p38 isoforms (Fig. 3B); therefore, REX1 knocked-down hUCB-MSCs were treated with BIRB796. After p38 MAPK inhibitor treatment, the level of Pp38 in REX1 knocked-down hUCB-MSCs was not different compared to vehicle control-infected hUCB-MSCs (Fig. 3C and 3E). After BIRB796 treatment, the cell proliferation defect of REX1 knocked-down hUCB-MSCs recovered, and the proliferation rate was similar to the rate in vehicle control-infected hUCB-MSCs (Fig. 3D). Similarly, in hUCB-MSCs, 1 µM or 10 µM BIRB796 treatment repressed the phosphorylation of p38 MAPK (Fig. 3E). In REX1 knocked-down hUCB-MSCs, the suppressed expression levels of CDK2 and CCND1 recovered after BIRB796 treatment (Fig. 3F). The expression of p53 and hyper-phosphorylated RB (PpRB) did not change in REX1 knocked-down cells (Fig. 3F). In hUCB-MSCs, the expression levels of ERK1/2 and Mitogen-Activated Protein Kinase Kinase 1/2 (MAP2K1/2 or MEK1/2) did not change after the knockdown of REX1. The phosphorylation of MEK (pMEK1/2) and NF-κB expression were not significantly different after the knockdown of REX1 in hUCB-MSCs compared to vehicle control-infected hUCB-MSCs (Fig. 3G). REX1 inhibits signaling via the STAT pathway in F9 cells [12]; however, the expression of STAT5 did not significantly change after REX1 knockdown in hUCB-MSCs. The expression levels of STAT3 and phosphorylated STAT3 increased after REX1 knockdown without regard to p38 MAPK inhibitor treatment (Fig. 3H). After REX1 knockdown in hUCB-MSCs, the expression levels of the p38s did not significantly change (Fig. 3I). In addition, BIRB 796 treatment did not influence REX1 expression in hUCB-MSCs (Fig. 3J).

### MKK3 expression significantly increased after REX1 knockdown

**Figure 4:**
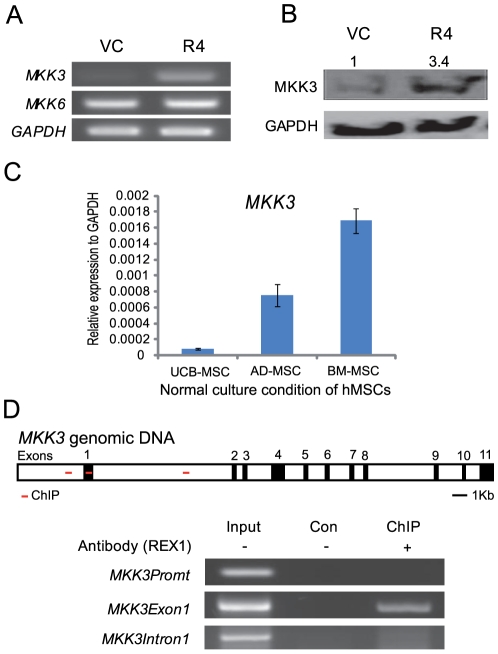
The expression of MKK3/6 in REX1 knocked-down hUCB-MSCs and normally cultured hMSCs, and the *MKK3* ChIP assay in hUCB-MSCs. (A) *MKK3* expression significantly increased, but *MKK6* did not significantly change after REX1 knockdown as shown by RT-PCR. (B) MKK3 increased after REX1 knockdown at the protein expression level. (C) The expression level of *MKK3* in hUCB-MSCs was 20-fold less than the level in hBM-MSCs under normal culture conditions as shown by real-time RT-PCR. (D) The ChIP assay for REX1. Three regions have REX1 consensus sequences in the *MKK3* genomic DNA. REX1 binds to the first exon region (MKK3Exon1) of *MKK3*. Abbreviations: MKK3Promt, MKK3 promoter region; MKK3Exon1, MKK3 exon 1 region; MKK3Intron1, MKK3 intron 1 region. Error bars represent the standard deviation from three independent experiments.

In mammals, MKK3 and MKK6 are well known activators of p38 MAPK via the phosphorylation of p38 MAPK [33]. The expression of *MKK6* did not significantly change after the knockdown of REX1, but the expression of *MKK3* dramatically increased in REX1 knocked-down hUCB-MSCs (Fig. 4A). The *MKK3* RNA level increased approximately seven-fold as confirmed by real-time RT-PCR. The level of MKK3 protein expression also increased 3.4-fold (Fig. 4B). In hMSCs, the expression of *MKK3* was in inverse proportion to REX1 expression (Figs. 1A and 4C). The expression level of *MKK3* in hUCB-MSCs was 20-fold less than the level in hBM-MSCs. Therefore, the direct regulation of *MKK3* by REX1 was investigated. The human genomic DNA sequences of *MKK3* were analyzed for the presence of the REX1 binding motif. The DNA binding motif of REX1 contains the core sequences of GCAGCCAT or GCCATTA [34]. In human genomic DNA, only three positions in *MKK3* contain consensus sequences for REX1 binding. These consensus sequences are located 1 Kb upstream of the first exon (Promoter region), inside of the first exon (Exon 1) and inside of the first intron (Intron 1) (Fig. 4D). A ChIP assay was performed to confirm the direct binding of REX1 to *MKK3*, and REX1 specifically binds only to the first exon of *MKK3* (Fig. 4D). Other regions of *MKK3* did not contain REX1 binding motifs or a positive signal from the ChIP assay.

### Alterations in the differentiation ability, and NOTCH and WNT signaling, of hUCB-MSCs following REX1 knockdown

**Figure 5:**
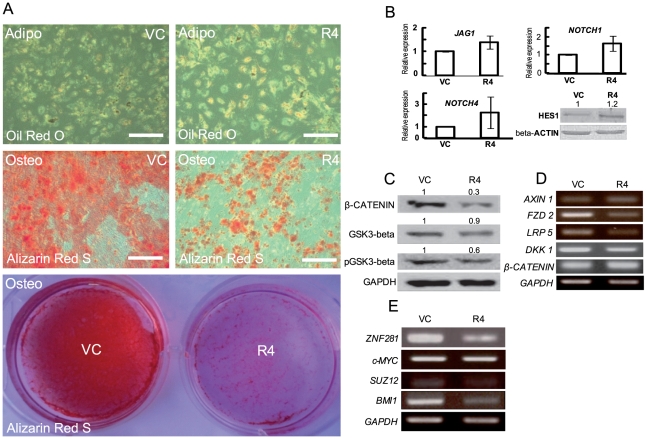
Differentiation study, NOTCH and WNT expression changes in REX1 knocked-down hUCB-MSCs. (A) After a three week induction, adipogenic and osteogenic differentiated hUCB-MSCs were stained with Oil Red O or Alizarin Red S. The number of adipogenic differentiated cells was similar to vehicle control-infected hUCB-MSCs or was slightly increased in REX1 knocked-down hUCB-MSCs. Osteogenesis of REX1 knocked-down hUCB-MSCs decreased compared to vehicle control-infected hUCB-MSCs. (B) The expression changes of NOTCH signaling genes after REX1 knockdown. The expression levels of *JAG1*, *NOTCH1* and *NOTCH4* increased after REX1 knockdown. HES1 expression increased 1.2-fold after REX1 knockdown. (C) The expression levels of GSK3-β, phospho-GSK3-β at serine-9 (pGSK3β) and β-CATENIN decreased after REX1 knock-down in hUCB-MSCs. (D) The expression levels of *FZD2*, *LRP5* and *DKK1* decreased after REX1 knockdown in hUCB-MSCs. (E) The expression levels of core transcription factors and polycomb group genes. The expression level of *ZNF28*1 was down-regulated after REX1 knockdown in hUCB-MSCs. The expression levels of *SUZ12* and *BMI1* also decreased after REX1 knockdown. The expression of *c-MYC* did not change after REX1 knockdown. Abbreviations: Adipo, adipogenic induction; Osteo, osteogenic induction. Error bars represent the standard deviation from three independent experiments. Scale bars represent 100 µm.

The differentiation ability of hUCB-MSCs was investigated after the knockdown of REX1. The adipogenic potentiality in REX1 knocked-down hUCB-MSCs slightly increased or was similar to vehicle control-infected hUCB-MSCs after adipogenic differentiation as shown by Oil Red O staining. However, the osteogenic differentiation potential of REX1 knocked-down hUCB-MSCs visibly deteriorated after osteogenic induction as shown by Alizarin Red S staining (Fig. 5A).

Notch signaling is important for the differentiation of MSCs [35], [36]. JAGGED1 (JAG1) is a ligand of the NOTCH receptor and plays key roles in cell differentiation and morphogenesis [37]. The expression of *JAG1* was up-regulated in REX1 knocked-down hUCB-MSCs compared to vehicle control-infected hUCB-MSCs. NOTCH proteins are single-pass trans-membrane receptors that regulate cell fate decisions during development. The expression levels of *NOTCH1* and *NOTCH4* also increased in REX1 knocked-down hUCB-MSCs compared to vehicle control-infected hUCB-MSCs (Fig. 5B). As a consequence, the expression of HES1, a NOTCH target molecule, was up-regulated in REX1 knocked-down hUCB-MSCs compared to vehicle control-infected hUCB-MSCs (Fig. 5B).

Canonical WNT/β-CATENIN signaling also plays an important role in regulating the differentiation of MSCs [38], [39]. The expression levels of β-CATENIN, GSK3-β and phospho-GSK3-β at serine-9 decreased after REX1 inhibition in hUCB-MSCs (Fig. 5C). The expression of a WNT signaling related gene, *AXIN1*, was not significantly different in REX1 knocked-down and vehicle control-infected hUCB-MSCs; however, the expression levels of *FZD2*, *LRP5* and *DKK1* decreased in REX1 knocked-down hUCB-MSCs compared to vehicle control-infected hUCB-MSCs (Fig. 5D). The expression of *ZNF281*, a core transcription factor in stem cells that functions during hMSC osteogenesis (unpublished data), decreased in REX1 knocked-down hUCB-MSCs compared to the vehicle control-infected hUCB-MSCs (Fig. 5E). The expression of *c-MYC*, another core transcription factor of stem cells, did not change significantly after REX1 knockdown in hUCB-MSCs. In order to evaluate the changes in expression of other polycomb group genes, the expression levels of two polycomb group genes that are important to maintenance and specification of stem cells [40], *SUZ12* and *BMI1*, were measured. The expression levels of *SUZ12* and *BMI1* decreased after REX1 knockdown in hUCB-MSCs (Fig. 5E).

### REX1 inhibition did not affect apoptosis of hUCB-MSCs and Pp38 level was not significantly altered after REX1 knockdown in hBM-MSCs

**Figure 6:**
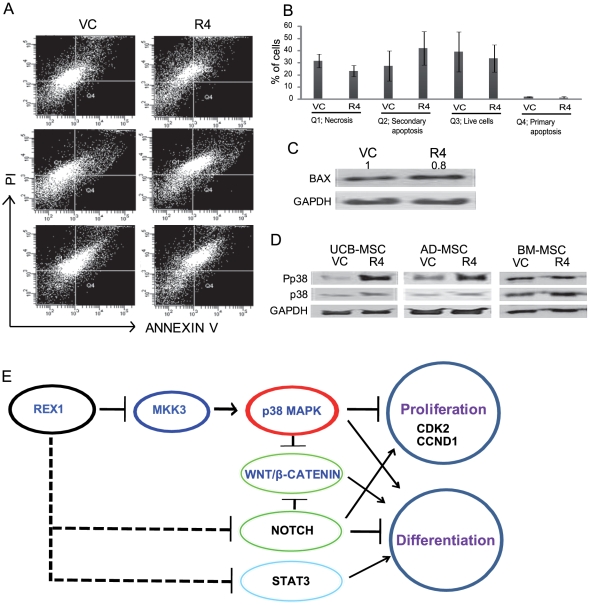
Apoptosis after REX1 knockdown in hUCB-MSCs and REX1 inhibition in hMSCs and the summary of the role of REX1 in stem cells. (A, B) Apoptotic cell death was not significantly different in REX1 knocked-down and vehicle control-infected hUCB-MSCs. (C) The expression of BAX did not change after REX1 knockdown in hUCB-MSCs. (D) Significant activation of p38 MAPK was observed after REX1 knockdown in hUCB-MSCs and hAD-MSCs but only slightly increased in hBM-MSCs. hBM-MSCs, which have low expression of REX1, have highly activated p38 MAPK (Pp38) in vehicle control-infected cells. (E) REX1 suppresses MKK3 expression, which activates p38 MAPK. REX1 also suppresses STAT3 expression and NOTCH signals. Error bars represent the standard deviation from three independent experiments.

Apoptosis is influenced by p38 MAPK, which is also important to cell growth. ANNEXIN V is used as a probe in the ANNEXIN V assay to detect cells that express phosphatidyl serine on the cell surface, a feature found in apoptosis as well as other forms of cell death [41], [42]. ANNEXIN V staining was performed on three hUCB-MSCs clones derived from three different individuals (Fig. 6A). The number of apoptotic cells (Q2 and Q4 fraction) or necrotic cells (Q1 fraction) of REX1 knocked-down hUCB-MSCs did not significantly differ from the vehicle control-infected hUCB-MSCs (Fig. 6B). The expression of BAX, a marker protein of apoptosis, also did not increase in REX1 knocked-down hUCB-MSCs compared to vehicle control-infected hUCB-MSCs (Fig. 6C).

In hAD-MSCs, the level of Pp38 significantly increased after the knockdown of REX1 similar to hUCB-MSCs. However, in hBM-MSCs, the level of Pp38 increased slightly after REX1 knockdown (Fig. 6D). Basically, in hBM-MSCs, REX1 expression was very low (Fig. 1A). Also, under normal culture conditions, the intrinsic Pp38 and MKK3 expression levels were much higher in hBM-MSCs than in hUCB-MSCs or in hAD-MSCs (Figs. 1B, 4C and 6D). Therefore, the effect of the REX1 knockdown did not significantly influence Pp38 level in hBM-MSCs compared to other types of hMSCs.

## Discussion

The p38 MAPK is important for cell growth, apoptosis and differentiation in mammalian cells. Under specific culture conditions, in hUCB-MSCs, which strongly express REX1, p38 MAPK is suppressed. However, hBM-MSCs, which have weak REX1 expression, have activated p38 MAPK and a high expression level of MKK3 under normal culture conditions. REX1 expression was inversely correlated with p38 MAPK activation and positively correlated with the proliferation rates of the three types of hMSCs examined.

REX1, a pluripotent marker gene, was expressed in some types of hMSCs and cancer cells as well as ESCs. In hUCB-MSCs, the knockdown of REX1 resulted in severe growth retardation. The functions of p38α have been previously reported and p38α is a major component of p38 MAPK in the majority of cells. p38α can negatively regulate cell cycle progression at both the G1/S and G2/M transitions by several mechanisms such as, the down-regulation of Cyclins and the up-regulation of CDK inhibitors [43]. In REX1 knocked-down hUCB-MSCs, the cell cycle was arrested primarily in the G0/G1 stage, compared to vehicle control-infected hUCB-MSCs. Passage through the cell cycle requires the successive activation of different cyclin-dependent protein kinases (CDKs) and Cyclins [44], [45]. Cyclin E, in association with CDK2, is required for the G_1_/S transition [46], [47]. Both Cyclin A and the B-type Cyclins associate with CDC2 to promote entry into mitosis [48]. The expression levels of CDK2, CCND1 and Cyclin B1 decreased in REX1 knocked-down hUCB-MSCs compared with the levels in vehicle control-infected hUCB-MSCs. The expression levels of CDK2 and CCND1 recovered after p38 MAPK inhibitor treatment in REX1 knocked-down hUCB-MSCs. However, the expression levels of CDK inhibitors, p53 and PpRB, did not change after p38 MAPK inhibitor treatment or REX1 knock-down in hUCB-MSCs. Although p38 MAPK was highly activated in REX1 knocked-down hUCB-MSCs, the cells grew slowly. Notch signaling, which increased in REX1 knocked-down hUCB-MSCs, also regulates stem cell number and has an opposite role of p38 MAPK [49]. Therefore, in REX1 knocked-down hUCB-MSCs, the growth inhibition signal by activated p38 MAPK was partially compensated by the cell proliferation signals from NOTCH activation.

Activation of p38 MAPK also contributes to chemically-induced cell death [50]. The possibility of increasing apoptosis in REX1 knocked-down hUCB-MSCs was excluded because the results of the ANNEXIN V assay showed no significant difference between REX1 knocked-down and vehicle control-infected hUCB-MSCs. In addition, the expression of STAT3 increased in REX1 knocked-down hUCB-MSCs, which was also previously reported [12]. After p38 MAPK inhibitor treatment and REX1 knockdown, the STAT3 activation patterns did not change, suggesting that STAT3 suppression by REX1 is independent of p38 MAPK signaling.

Activation of p38 MAPK is important for the differentiation of mouse ESCs [51] and hMSCs [52]. The known up-stream regulators of p38 MAPK are MKK3 and MKK6, which are activated in response to many types of cell stresses [53], [54]. However, in stem cells, the transcriptional regulation of *MKK*3 and *MKK6* was not previously reported. REX1 expression is specific to pluripotent stem cells, several types of hMSCs and cancer cells. REX1 is highly expressed in hUCB-MSCs, but *MKK3* expression is approximately 20-fold lower in hUCB-MSCs, compared to hBM-MSCs, where REX1 expression is low under normal culture conditions. REX1 suppresses the expression of MKK3 in hMSCs, which was mediated by REX1 binding directly to the first exon of *MKK3* (Fig. 6E).

The Notch signaling pathway is implicated in the differentiation [55], [56] and the immune-modulation of MSCs [57]. Notch signaling inhibits chondrogenesis of hMSCs [35]. Also, the expression of the NOTCH ligand and its receptors (JAG1, NOTCH1 and NOTCH 4) increased after the knockdown of REX1 in hUCB-MSCs. Therefore, osteogenic differentiation deformity was partially caused by NOTCH activation after REX1 knockdown in hUCB-MSCs. Notch signaling also has a role in adipogenesis, and in 3T3-L1 cells, the reduction of Hes-1 inhibits adipogenic differentiation [58]. The activation of p38 MAPK activates C/EBPβ, a transcription factor that has a critical role in adipogenesis [59]. Therefore, after REX1 knockdown in hUCB-MSCs, increased adipogenesis was potentially caused by both NOTCH and p38 MAPK activation. The expression level of HES1, a target molecule of NOTCH signaling, increased in REX1 knocked-down hUCB-MSCs; therefore, REX1 may suppress the NOTCH signaling pathways and maintains the undifferentiated state of hUCB-MSCs (Fig. 6E).

In MSCs, Wnt/β-catenin (i.e., canonical) signaling is also important to maintain stemness and multipotency [60]–[62]. β-CATENIN decreased after REX1 knockdown in hUCB-MSCs. The function of GSK3-β, which inactivates β-CATENIN, is inhibited by the phosphorylation of serine-9 [63]. In REX1 knocked-down hUCB-MSCs, the expression level of GSK3-β decreased, and the level of phosphorylated GSK3**-**β at serine-9 also decreased. p38 MAPK down-regulates WNT/β-CATENIN signaling via GSK3-β [64], which was confirmed by our results. Notch 1 over-expression inhibits osteoblastogenesis by suppressing Wnt/β-Catenin signaling [65], which correlated with osteogenic deformity in REX1 knocked-down hUCB-MSCs. The increased NOTCH signal also caused the suppression of WNT in REX1 knocked-down hUCB-MSCs. Several core transcription factors that have critical roles in ESCs were expressed in hUCB-MSCs. The effect of REX1 knockdown on these transcription factors was different in each gene expression. The expression of *ZNF281* decreased in REX1 knocked-down hUCB-MSCs compared to vehicle control-infected hUCB-MSCs, but the expression of *c-MYC* did not change when REX1 was knocked-down in hUCB-MSCs. REX1 is considered to be a member of the YY1 gene family which is a polycomb group gene. The expression levels of the polycomb group genes, *SUZ12* and *BMI1*, were reduced in REX1 knocked-down hUCB-MSCs. Therefore, REX1 also has a role in modulating the chromatin structure of hUCB-MSCs, which needs to be further explored.

In conclusion, REX1 represses the expression of MKK3, which can activate p38 MAPK in hMSCs. The high expression level of REX1 in stem cells protects cell differentiation by suppressing p38 MAPK activation via MKK3 suppression and by suppressing NOTCH and STAT3 signaling. ASCs that highly express REX1 have more prominent cell-proliferation ability and multipotency than low REX1-expressing ASCs due to the p38 MAPK suppression in stem cells. Therefore, REX1 is a proper marker not only in ESCs but also in highly efficient multipotent human ASCs. Overall, the suppression of p38 MAPK is useful in the collection and culturing of multipotent human ASCs that do not have sufficient expression levels of REX1. This will be beneficial to the field of regenerative medicine.

## Supporting Information

Table S1 PCR primers sequences used in experiment.
(0.12 MB DOC)
