# Supplementary material for: REX-1 Expression and p38 MAPK Activation Status Can Determine Proliferation/Differentiation Fates in Human Mesenchymal Stem Cells
Source: PLoS One. 2010 May 5;5(5):e10493. doi: 10.1371/journal.pone.0010493 (PMC2864743; doi:10.1371/journal.pone.0010493)
Supplement: Table S1 — PCR primers sequences used in experiment. (0.12 MB DOC) [file pone.0010493.s001.doc]

**Table S1. PCR primers sequences used in experiment**

| Gene Primer sequence References | | |
| --- | --- | --- |
| REX1 | F 5′-GCGTACGCAAATTAAAGTCCAGA-3′ | [1] |
|  | R 5′-CAGCATCCTAAACAGCTCGCAGAAT-3′ |  |
| ZNF281 | F 5′-ACGTAACAGCGCAGACAGAA-3′ | [2] |
|  | R 5′-GTGTTGAAGCCCAAGTGGTT-3′ |  |
| c-MYC | F 5′-TACCCTCTCAACGACAGCAG-3′ | [3] |
|  | R 5′-GGGCTGTGAGGAGGTTTG-3′ |  |
| SUZ12 | F 5′-TGGGAGACTATTCTTGATGG-3′ | [4] |
|  | R 5′-GGAGCCGTAGATTTATCATT-3′ |  |
| BMI-1 | F 5′-GGAGACCAGCAAGTATTGTCCTTTTG-3′ | [5] |
|  | R 5′-CATTGCTGCTGGGCATCGTAAG-3′ |  |
| JAG1 | F 5′-GCTGGCAAGGCCTGTACTG-3′ | [6] |
|  | R 5′-ACTGCCAGGGCTCATTACAGA-3′ |  |
| NOTCH 1 | F 5′-CGGGTCCACCAGTTTGAATG-3′ | [6] |
|  | R 5′-GTTGTATTGGTTCGGCACCAT-3′ |  |
| NOTCH 4 | F 5′-CGGCCTCGGACTCAGTCA-3′ | [6] |
|  | R 5′-CAACTCCATCCTCATCAACTTCTG-3′ |  |
| AXIN1 | F 5′-TGATAACAATGGCATCGTGTCC-3′ | [7] |
|  | R 5′-GTCCTGGTCACACTTCCATTCC-3′ |  |
| FZD2 | F 5′- CACGCCGCGCATGTC-3′ | [8] |
|  | R 5′- ACGATGAGCGTCATGAGGTATTT-3′ |  |
| LRP5 | F 5′-GACCCAGCCCTTTGTTTTGAC-3′ | [8] |
|  | R 5′-TGTGGACGTTGATATTGGT-3′ |  |
| DKK1 | F 5′-AACCAGCTATCCAAATGCAG-3′ | [9] |
|  | R 5′-TCACAGGGGAGTTCCATAAA-3′ |  |
| β-CATENIN | F 5′-TCTGATAAAGGCTACTGTTGGATTGA-3′ | [10] |
|  | R 5′-TCACGCAAAGGTGCATGATT-3′ |  |
| MKK3 | F 5’-GTCCAAGCCACCCGCAC-3’ | [11] |
|  | R 5’-CCTCAAAGTTTCTGTCTCCAATGG-3’ |  |
| MKK6 | F 5’-GAACTGGGACGAGGTGCGTA-3’ | [11] |
|  | R 5’-TTTACTGTGGCTCGGATCCG-3’ |  |
| MKK3 ChIP (promoter) | F 5’-GCCTGGTACATTCTGCCTTC-3’ | N/A |
|  | R 5’-CAGTCTGCCCATCTGCATAA-3’ |  |
| MKK3 ChIP (first exon) | F 5’-TGGAAACGAAAGGACCAATC-3’ | N/A |
|  | R 5’-GACGGCGGTGGAGACTAAT-3’ |  |
| MKK3 ChIP (first intron) | F 5’-CACAAGTGGTTCCATGTTGC-3’ | N/A |
|  | R 5’-GGGATCCCCAGACTATCCAC-3’ |  |
| p 38 α | F 5’-GTGCCCGAGCGTTACCAGAAC-3’ | [12] |
|  | R 5’-CTGTAAGCTTCTGACATTTC-3’ |  |
| p 38 β | F 5’-CACCCAGCCCTGAGGTTCT-3’ | [12] |
|  | R 5’-AATCTCCAGGCTGCCAGG-3’ |  |
| p 38 γ | F 5’-ACATGAAGGGCCTCCCCG-3’ | [12] |
|  | R 5’-TCTCCTTGGAGACCCTGG-3’ |  |
| p 38 δ | F 5’-CCCAAGACCTACGTGTCCC-3’ | [12] |
|  | R 5’-ACTGGATCTTCTCCTCACTG-3’ |  |
| GAPDH | F 5′-GAGTCAACGGATTTGGTCGT-3′ | [13] |
|  | R 5′-GACAAGCTTCCCGTTCTCAG-3′ |  |

N/A, not applicable

**SUPPLEMENTAL REFERENCES**

1. Miki T, Lehmann T, Cai H, Stolz DB, Strom SC (2005) Stem cell characteristics of amniotic epithelial cells. Stem Cells 23: 1549-1559.

2. http;//www.ncbi.nlm.nih.gov/genome/sts/sts.cgi?uid=9777.

3. http;//www.ncbi.nlm.nih.gov/genome/sts/sts.cgi?uid=266881.

4. Bracken AP, Kleine-Kohlbrecher D, Dietrich N, Pasini D, Gargiulo G, et al. (2007) The Polycomb group proteins bind throughout the INK4A-ARF locus and are disassociated in senescent cells. Genes Dev 21: 525-530.

5. Pasquinelli G, Tazzari PL, Vaselli C, Foroni L, Buzzi M, et al. (2007) Thoracic aortas from multiorgan donors are suitable for obtaining resident angiogenic mesenchymal stromal cells. Stem Cells 25: 1627-1634.

6. Yu LM, Chen DX, Zhou QX, Fang N, Liu ZL (2006) Effects of histamine on immunophenotype and notch signaling in human HL-60 leukemia cells. Exp Biol Med (Maywood) 231: 1633-1637.

7. Yordy JS, Moussa O, Pei H, Chaussabel D, Li R, et al. (2005) SP100 inhibits ETS1 activity in primary endothelial cells. Oncogene 24: 916-931.

8. Konigshoff M, Balsara N, Pfaff EM, Kramer M, Chrobak I, et al. (2008) Functional Wnt signaling is increased in idiopathic pulmonary fibrosis. PLoS One 3: e2142.

9. http://www.realtimeprimers.com.

10. Suriano G, Vrcelj N, Senz J, Ferreira P, Masoudi H, et al. (2005) beta-catenin (CTNNB1) gene amplification: a new mechanism of protein overexpression in cancer. Genes Chromosomes Cancer 42: 238-246.

11. Junttila MR, Ala-Aho R, Jokilehto T, Peltonen J, Kallajoki M, et al. (2007) p38alpha and p38delta mitogen-activated protein kinase isoforms regulate invasion and growth of head and neck squamous carcinoma cells. Oncogene 26: 5267-5279.

12. Uddin S, Ah-Kang J, Ulaszek J, Mahmud D, Wickrema A (2004) Differentiation stage-specific activation of p38 mitogen-activated protein kinase isoforms in primary human erythroid cells. Proc Natl Acad Sci U S A 101: 147-152.

13. Valbuena D, Galan A, Sanchez E, Poo ME, Gomez E, et al. (2006) Derivation and characterization of three new Spanish human embryonic stem cell lines (VAL -3 -4 -5) on human feeder and in serum-free conditions. Reprod Biomed Online 13: 875-886.
